# Modeling the behavior of monoclonal antibodies on hydrophobic interaction chromatography resins

**DOI:** 10.1186/s40643-024-00738-8

**Published:** 2024-02-15

**Authors:** Douglas Nolan, Thomas R. Chin, Mick Eamsureya, Sheldon Oppenheim, Olga Paley, Christina Alves, George Parks

**Affiliations:** 1 Takeda Pharmaceuticals America Inc, Lexington, MA 02421 USA; 2 Eurofins Lancaster Laboratories Professional Scientific Services, LLC, Lancaster, PA 17601 USA

**Keywords:** Bioprocess engineering, Chromatography, Statistical analysis, Bioengineering

## Abstract

Monoclonal antibodies (mAbs) require a high level of purity for regulatory approval and safe administration. High-molecular weight (HMW) species are a common impurity associated with mAb therapies. Hydrophobic interaction chromatography (HIC) resins are often used to remove these HMW impurities. Determination of a suitable HIC resin can be a time and resource-intensive process. In this study, we modeled the chromatographic behavior of seven mAbs across 13 HIC resins using measurements of surface hydrophobicity, surface charge, and thermal stability for mAbs, and hydrophobicity and zeta-potential for HIC resins with high fit quality (adjusted *R*^2^ > 0.80). We identified zeta-potential as a novel key modeling parameter. When using these models to select a HIC resin for HMW clearance of a test mAb, we were able to achieve 60% HMW clearance and 89% recovery. These models can be used to expedite the downstream process development for mAbs in an industry setting.

**Graphical Abstract:**

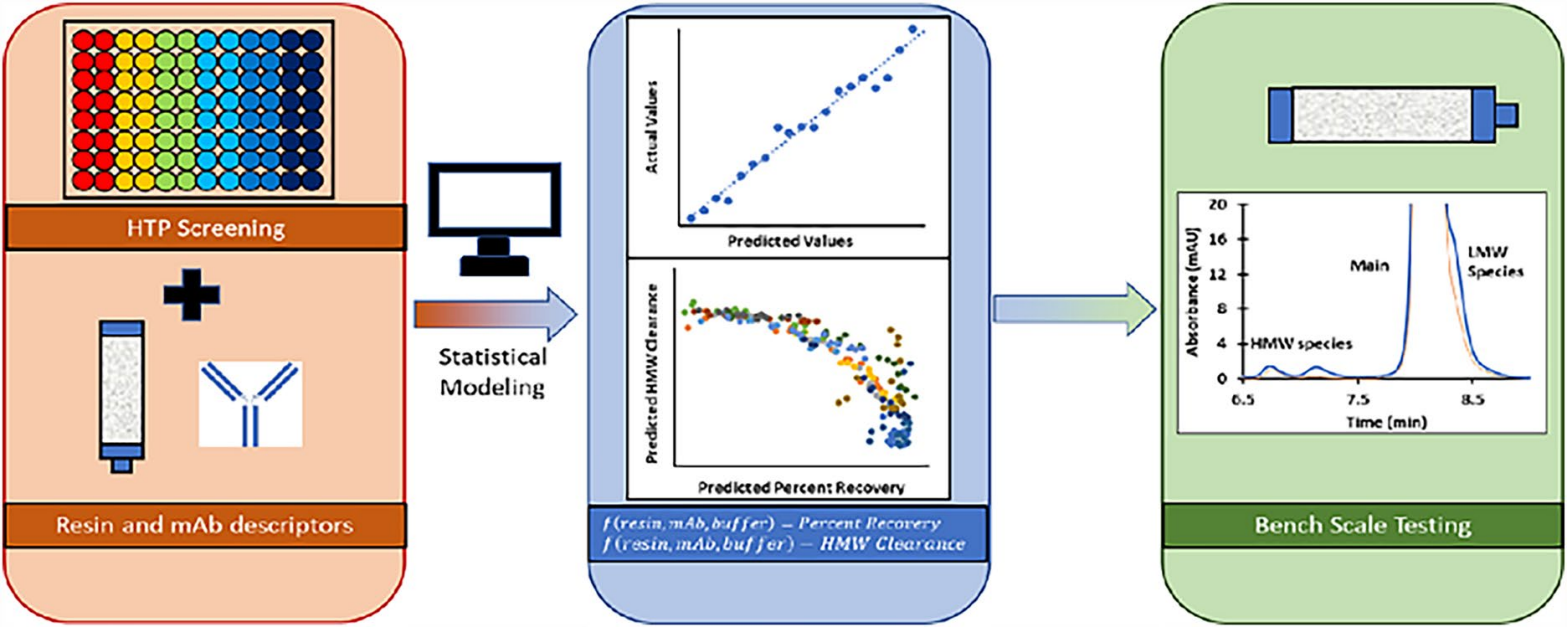

**Supplementary Information:**

The online version contains supplementary material available at 10.1186/s40643-024-00738-8.

## Introduction

Monoclonal antibodies (mAbs) are a well-established class of biotherapeutics (Lyu et al. 2022; Haraya et al. 2022). MAb therapies require a high-level of purity for regulatory approval and commercialization. Typical industry downstream processes consist of an affinity capture step followed by 2–3 polishing steps [anion-exchange (AEX) chromatography, cation exchange (CEX) chromatography, multi-modal chromatography (MMC), and/or hydrophobic interaction chromatography (HIC)] to bring the level of impurities (high molecule weight (HMW) aggregates, host cell proteins (HCPs), DNA, etc.) down to acceptable levels. 

High molecular weight aggregates are a common impurity resulting from mAb instability. These HMW aggregates can remain at clinically relevant levels even after the ProA affinity capture step. Within the biopharmaceutical industry, mAb aggregates are usually defined as any multimeric species arising from the reversible or irreversible association of monomer species (Mahler et al. 2009). Reversible aggregation results from the non-covalent self-association of monomer species and is mediated by buffer composition (pH, ionic strength, protein concentration, etc.). Irreversible formation of aggregates can occur either by covalent interactions (e.g., disulfide bonds) or by conformational changes that result from partial unfolding of the mAb followed by exposure of the hydrophobic patches. The exposed hydrophobic patches associate to form aggregate species. Bioreactor agitation, exposure to liquid–air interfaces, and shear related effects during upstream processing and downstream material processing can also contribute to the irreversible formation of aggregates by inducing mAb unfolding and subsequent aggregation. Retention of these aggregate impurities in final drug substance formulation can increase immunogenicity (Singh 2011) and decrease potency (Paul et al. 2012). Therefore, the final drug product must contain low levels of HMW species for safe administration.

HIC chromatography is a common process step used in downstream processing to remove HMW species. While SEC can be used to remove HMW species, the low flowrates (< 50 cm/h) and loading capacity make it unsuitable for removal of HMW species on a manufacturing scale. HIC separates the target of interest from impurities based on differences in hydrophobic characteristics. In the context of HMW clearance, the increased mass and size of aggregates leads to a greater hydrophobic and total surface area that allows for increased binding to the resin surface. Thus, HIC steps are typically performed in flow-through mode in which the monomer passes through the resin while the aggregates are retained.

The retention of mAbs and their separation from HMW impurities results from a complex buffer–resin–mAb interaction. The behavior of a mAb on a resin is dependent on its own properties (e.g., hydrophobicity, surface charge, stability, etc.) as well as the properties of the resin (ligand type, ligand density, support, particle size, etc.). The buffer composition can also affect the properties of both the mAb and the resin and how they interact with each other. Interactions of a protein with a hydrophobic surface can lead to conformational changes (Ueberbacher et al. 2008; Beyer and Jungbauer 2018; Jungbauer et al. 2005) and subsequent aggregation. HIC conditions must therefore be carefully adjusted to account for both high percent recovery of the monomer species and high HMW clearance.

There are several commercially available HIC resins and determining the optimal HIC resin for HMW clearance and percent recovery can be time and resource intensive. While HTS (High Throughput Screening) and DOE (Design of Experiments) techniques exist to rapidly screen resin–buffer conditions, it would be ideal to select the optimal resin–buffer condition based on a few measurements of the protein characteristic without having to perform extensive HTS experiments. Such predictive models, based on measurable physicochemical properties of mAbs and potential resins, would drive the understanding of important properties of mAbs and resins, and how the two interact. These models could then be used to expedite the development of purification processes of these mAbs.

Quantitative-Structure Activity Relationship (QSAR) modeling is a technique applied to predict biological activity of small molecules (Hansch et al. 1962). The application of QSAR has extended beyond its use in drug discovery to cover a diverse range of areas (Muratov et al. 2020) including predicting the behavior of proteins on different chromatographic media [CEX (Mazza et al. 2001; Malmquist et al. 2006; Robinson et al. 2017), AEX (Malmquist et al. 2006; Song et al. 2002), HIC (Robinson et al. 2017; Ladiwala et al. 2006) and MMC (Robinson et al. 2017; Woo et al. 2015; Hou and Cramer 2011)]. Development of high-throughput plate (HTP) technology has allowed for the rapid screening of proteins across several resin and buffer conditions for the prediction of column performance (Kramarczyk et al. 2008; Nfor et al. 2011; Coffman et al. 2008). The ability for these HTP techniques to rapidly screen protein across different resin and buffer conditions makes it a useful tool for collecting the large data sets needed for QSAR modeling. In this study, seven mAbs (five IgG1 and two IgG2s) were evaluated based on different physicochemical properties (surface hydrophobicity, surface charge, and thermal stability) and screened across several HIC resins and buffer conditions using an HTP technique. Measurements of the resin hydrophobicity and zeta-potential were also performed. Percent flow-through recovery and HMW clearance were measured for each mAb–resin–buffer condition. The results of the physicochemical evaluation were correlated with percent recovery and HMW clearance data to generate a predictive model to identify a suitable HIC resin for the purification of mAbs using HIC chromatography. Finally, the model was used to predict an optimal resin for a test mAb using small-scale chromatography condition.

### Materials

All chemicals were purchased from commercial vendors.

Pre-packed columns and bulk resins of POROS Ethyl, POROS Benzyl, and POROS Benzyl Ultra columns were obtained from ThermoScientific. Pre-packed columns and bulk resins for TOYOPearl PPG 600 M, TOYOPearl Phenyl 600 M, and TOYOPearl Butyl 600 M were purchased from TOSOH. Pre-packed columns, pre-packed 96-well plates, and bulk resins for Capto Phenyl ImPres, Capto Butyl ImPres, Capto Octyl, Phenyl Sepharose FF LS, ButylS FF, Capto Butyl, Capto Butyl and Phenyl Sepharose FF HS were purchased from Cytiva.

TSKgel Butyl-NPR 4.6 mm ID × 3.5 cm, 2.5 µm was purchased from TOSOSH. CE capillary, µSIL-FC, 50 µm ID, 56 cm was purchased from Agilent. 

All mAbs were produced in Chinese Hamster Ovary (CHO) cells using a proprietary media and culture process. All mAbs were purified using an affinity Protein A capture step. MAb C had been further purified to the level of drug substance. MAb D was brought to pH 5 by the addition of 1 M trisodium citrate following the affinity capture step. The post-capture step material was stored at − 80 °C until further use. 

### Resin hydrophobicity measurements

Each column was equilibrated with at least 3 column volumes (CVs) of mobile phase buffer (50 mM sodium citrate, 350 mM ammonium sulfate pH 7). Samples were buffered exchanged into the mobile phase by repeated concentration and dilution using a centrifugal filter device (30 K Nominal Molecular Weight Cut-off NMWCO). Prior to analysis, samples were diluted to 20 mg/mL and injected onto the column using a 50 μL injection volume. Samples were eluted off the column using a 0.3 mL/min flow rate. Under our buffer conditions, mAb E took > 2 h to elute off these columns. Because of the resulting poor signal-to-noise ratio, mAb E was not included in this portion of the study.

The retention factor, *k*, was calculated using Eq. (1), where *t*_r_ is the retention time of the analyte and *t*_0_ is the retention time of an unretained tracer. For an analyte to accurately measure *t*_0_, the analyte should be approximately the same size as the molecule of interest. HIC resins can have an underlying size exclusion contribution to the separation of molecules (Huang et al. 2018). Attempts to use 150 kDa dextran resulted in strong retention on some of the test resins (TOYOPEARL Phenyl, Additional file 1: Fig. S1). To measure t_0,_ mAb B (the least hydrophobic mAb) was eluted through a column using a 25 mM sodium citrate, 25% *v*/*v* isopropanol pH 7 buffer. This method would only qualitatively assess t_0_ as we are not able to account for the change in void volume because of differences in mobile phase buffer:

1 $$k = \frac{{t_{{\mathrm{r}}} - t_{{0}} }}{{t_{{0}} }}.$$

### ***Streaming zeta-potential***

Zeta-potential analysis was performed by Material Characterization Services LLC using an Anton Paar SurPASS 3 Electrokinetic Analyzer with a 10 mM KCl electrolyte medium. Zeta-potential values for pH 5, 6, and 7 were interpolated using zeta-potential measurements of the pH values adjacent to the intended pH. The zeta-potential measurements are reported in Additional file 3: Table S1.

### Relative surface hydrophobicity (RSH)

RSH measurements were performed on a Water Alliance e2695 HPLC system. The TSKgel Butyl-NPR column was equilibrated with 25 mM sodium phosphate, 1.5 M ammonium sulfate, 0.025% sodium azide pH 7. Samples were eluted off the column using a 12-min linear gradient with 25 mM sodium phosphate, 0.025% sodium azide pH 7 at 1.0 mL/min flowrate. RSH is defined as the elution time divided by the total time of the gradient (12 min).

### Capillary zone electrophoresis (CZE) experiments

CZE experiments were performed on a Sciex PA 800 Plus instrument. The commercially available FC capillary was cut to a length of 30.2 cm with an inlet-detection window length of 20 cm. Two separation buffers were used in this study (a) 200 mM EACA, 0.05% HMPC pH 5 (b) and 400 mM EACA, 0.05% HPMC pH 6 were adapted from the literature (Moritz et al. 2015). The capillary was conditioned using 0.1 M NaOH (50 psi, 5 min) followed by the separation buffer (50 psi, 5 min). The column equilibrated by applying + 30 kV for 30 min.

Samples were diluted to 1 mg/mL using water. For each injection, the capillary was flushed with 0.1 M NaOH (30 psi, 0.5 min) followed by separation buffer (30 psi, 0.5 min). Samples were injected onto the column (0.5 psi, 10 s) and eluted using + 30 kV with a detection wavelength of 214 nm. After every 5–7 runs, the capillary was positioned into vials containing fresh separation buffer.

### Thermal stability measurements

Melting temperature (T_m_) and aggregation temperature (T_agg_) were measured by dynamic scanning fluorimetry (DSF) on an UNcle system (Unchained Labs). MAb samples were buffered exchanged into 50 mM Sodium Citrate at either pH 5, pH 6, or pH 7 by repeated dilution and concentration using a Centricon (30 K NMWCO) to final concentration of at least 25 mg/mL. The samples were diluted to 0.5 mg/mL into the appropriate buffer. Samples were transferred into an UNcle Uni cartridge. The melting temperature was measured using a linear 0.3 °C/min ramp rate from 25 to 95 °C. The T_m_ determined using the inflection point of the barycentric mean (BCM) vs temperature graph and the T_agg_ was determined by 10% height of the peak measuring the static light scattering (SLS) intensity at 266 nm.

### High-throughput plate (HTP) studies

Studies were conducted using a Tecan Evo200 instrument. All 96-well filter plates contained 50 μL of gravity settled resin in each well. MAb C was buffered exchanged into 50 mM sodium citrate pH 5 using a Pellicon 3 Biomax, 30 K NMWCO, C-screen, 88 cm^2^ membrane. MAb D was run through a MabSelect SuRe LX column to buffer exchange the material into 25 mM sodium citrate. Protein samples were diluted to 8 mg/mL pH and adjusted to either pH 5, pH 6, or pH 7 using 1 M NaOH. The samples were brought to the correct buffer and salt concentration using concentrated stocks of sodium citrate and sodium chloride or ammonium sulfate. Fifteen different buffer compositions consisting of 50 mM sodium citrate with either no salt addition, 250 mM sodium chloride or ammonium sulfate, and 500 mM sodium chloride or ammonium sulfate at either pH 5, 6, or 7 were screened in this study. The samples for each buffer condition were loaded into a deep well 96-well plate in triplicate.

The 96-well filter plates containing the resin were equilibrated with desired buffer by first evacuating the storage solution via centrifugation (3000 rpm for 5 min). Each of the wells was filled with 200 μL of the appropriate buffer, shaken for 3 min at 1100 rpm using a TeShake orbital plate shaker and then centrifuged at 3000 rpm for 5 min. After equilibration, 200 μL of the corresponding load material was dispensed into each of the wells, shaken for 3 min at 1100 rpm using an orbital plate shaker and centrifuged (3000 rpm, 5 min). This process was repeated three more times with respective equilibration buffer to wash the resin of any remaining protein. After washing the resin with equilibration buffer, a water strip step followed by a 0.1 M NaOH step was performed using the same process to remove any remaining protein. Concentrations were determined using a 96-well plate reader (Magellan, NC, USA). Protein absorption was measured at 280 nm and was corrected for the absorption of plastic 96-well plate by measuring the absorption at 340 nm. The pathlength was determined by measuring the absorption of water at 998 nm correcting for the absorption of plastic at 900 nm. Triplicate measurements were averaged together and when the RSD was greater than 10% the value furthest away from the average was removed.

### SEC analysis

SEC analysis for mAb B, mAb C, mAb D, mAb C, mAb F, and mAb G were measured using a UPLC-SEC method on a ThermoFisher Ultimate 3000 UPLC instrument. The SEC column (Water BEH SEC 200 4.6 mM × 30 cm, 1.7 μM particle size) was equilibrated with mobile phase (50 mm sodium phosphate, 200 mM sodium perchlorate pH 6.5). The sample injection volume was adjusted so that 20 μg of protein was injected onto the column equilibrated with the mobile buffer at 35 °C at 0.3 mL/min. The samples were detected at 280 nm.

The SEC analysis for mAb A was performed using a tandem-SEC method on a Water Alliance e2695 HPLC system. The guard column (TOSOSH Biosep, TSK Gel, Guard SWXL, 6 mm × 4 cm, 7 µm particle size) and two SEC columns (TOSOH Biosep, TSK Gel, G3000 SWXL) were equilibrated with mobile phase buffer (100 mM sodium phosphate, 300 mM sodium chloride, 10% *v*/*v* acetonitrile pH 7.0). Samples were diluted to 3.75 mg/mL with mobile phase buffer and 20 uL of sample were injected onto the column. In cases where sample concentration was lower than 3.75 mg/mL, the injection volume was adjusted to that 75 μg of protein was injected onto the column. The column temperature was held at 25 °C during the separation. Samples were eluted using a flowrate of 0.8 mL/min. Samples were detected at 280 nm.

### Small-scale chromatography

Small-scale chromatography experiments were performed on an AKTA 25 FPLC instrument. ProA eluate material of test mAb H was diluted to 8 mg/mL and pH adjusted to pH 5 using 1 M NaOH. The sodium citrate concentration was adjusted to 50 mM sodium citrate by the addition of 190 mM sodium citrate pH 5 to generate the HIC column load material.

A pre-packed 1 mL Capto Butyl HiTrap column was equilibrated with five CVs of 50 mM sodium citrate pH 5. A 5 mL capillary loop was filled with the mAb H load material. The load material was injected onto the column at a flowrate of 0.33 mL/min to achieve a residence time of 3 min. After the sample was applied, the column was washed with 50 mM sodium citrate pH 5 at a flow rate of 0.33 mL/min. Eluate collection occurred using 100 mAU ascending and descending peak collection criteria.

### JMP analysis

All models were fit with JMP 17.0.0 using the Fit Model application using a standard least square regression model. All models included pH, NaCl concentration, and ammonium sulfate concentration as main effects in addition to the main effects explicitly stated in the discussion of each model. The model effects are constructed using the in-built Response Surface macro. Once the model is generated, the least significant effect is iteratively removed to optimize the adjusted *R*^2^. Individual data points with a Studentized residuals outside the 95% simultaneous limits were removed.

## Results/discussion

Seven mAbs and 13 resins were used in this study to model percent recovery and HMW clearance across different buffer conditions. The mAb–resin adsorption process is largely mediated by the surface properties of each. Therefore, both surface hydrophobicity and surface charge were evaluated for both mAbs and resins used in this study. Additionally, measurements of T_m_ and T_agg_ onset were conducted to evaluate stability of each of the mAbs towards unfolding. The buffer conditions screened in this study cover a commonly used design space for downstream mAb processing.

### Resin hydrophobicity measurements

The hydrophobicity of a column is a complex mixture of ligand type, ligand density, ligand accessibility, and resin support. Other studies have used the elution time of a single molecule across different resins as a measurement of resin hydrophobicity (Jiang et al. 2010). However, the chosen molecule might not represent how all mAbs behave across HIC resins. Therefore, an alternative method was sought to account for variations in how different mAbs may interact across a selection of resins. Analysis of retention factors for a set of small molecules across different columns in the form of a ln k vs ln k have been used to assess hydrophobicity of RPLC columns (Snyder et al. 2004). In the case of HIC resins, the slope of ln k vs ln k plot is interpreted as measurement of the hydrophobicity of one resin relative to another. Our study used a smaller data set of molecules to generate the ln k vs ln k plots compared to other studies (Snyder et al. 2004). Therefore, resulting hydrophobicity measurement is interpreted as a qualitative evaluation of hydrophobicity of a column.

The retention factors for each mAb on each resin are summarized in Table 1. In our study, TOYOPearl Phenyl was the most hydrophobic resin used. Consequently, the ln k of each mAb on each resin was plotted against the ln k for that of TOYOPearl Phenyl as a reference to determine hydrophobicity values and correlation strength (Additional file 3: Table S1). Both Phenyl FF HS and POROS Benzyl Ultra could not be included in this analysis as the mAbs took more than two hours to elute off the resin resulting in poor signal-to-noise ratio. Each ln k vs ln k plot had a moderate to high correlation (*R*^2^) when plotted against TOYOPearl Phenyl.

**Table 1:** Retention factor values for each mAb A-F across different HIC resins

	mAb A	mAb B	mAb C	mAb D	mAb F	mAb G
POROS Ethyl	0.16	0.13	0.16	0.17	0.14	0.29
POROS Benzyl	1.00	0.39	0.89	1.06	0.61	4.15
TOYOPearl PPG	0.14	1.00	0.44	0.15	0.11	0.17
TOYOPearl Butyl	0.79	0.39	0.73	0.66	0.41	4.78
TOYOPearl Phenyl	2.79	0.85	3.58	1.92	1.71	15.87
Phenyl FF LS	0.38	0.29	0.59	0.45	0.43	0.91
Butyl S FF	0.12	0.10	0.12	0.14	0.13	0.32
Capto Octyl	0.25	0.23	0.24	0.23	0.23	0.38
Capto Butyl	0.75	0.57	0.95	4.33	0.56	1.99
Capto Butyl ImPres	0.35	0.24	0.33	0.31	0.29	0.97
Capto Phenyl ImPres	3.48	1.84	6.15	3.56	3.21	13.81

Retention factors were measured using a 50 mM sodium citrate 350 mM ammonium sulfate pH 7 mobile phase

While there was an overall high correlation strength across the different HIC resins, we did observe unique resin–mAb interactions. MAb D had an unexpectedly long retention on Capto Butyl (Table 1) and was not included in the ln k vs ln k plot of Capto Butyl. The unexpectedly long retention was not seen on Capto Butyl ImPres. The main difference between Capto Butyl and Capto Butyl ImPres is particle size (Additional file 3: Table S1), but it is unclear why the change in particle size could affect the retention of mAb D specifically. None of the other resins with similar particle size had unexpectedly long retention times (Additional file 3: Table S1). Another factor or combination of factors might contribute to this specific behavior of mAb D on Capto Butyl ImPres resin. MAb C exhibited an unexpectedly lower retention on POROS Benzyl. For aliphatic ligands, mAb C has a similar retention factor as mAb D but eluted about 40% longer in the aromatic resins (Additional file 3: Table S1) except for POROS Benzyl where mAb C had a similar retention factor as mAb D. The larger retention factor of mAb C relative to mAb D on aromatic ligands could be a result of increased cation–π interactions. Because of its higher pI (Table 2), mAb C is expected to have a greater positive charge on its surface compared to mAb D under the same buffer condition. It is unclear why mAb C does not exhibit longer retention times on POROS Benzyl resin. The aromatic ring ligand in POROS Benzyl is coupled to the resin via a methylene group while the other aromatic ligands connect to the resin support by an oxygen atom. While the through-space effects of these groups can affect the strength of the cation–π interactions (Ma and Dougherty 1997; Wheeler and Houk 2009a, 2009b), these effects are expected to be too small to explain these results.

**Table 2:** Properties of each mAb used in the study

mAb	Type	RSH	pI	CZE elution time pH 5 (min)	CZE elution time pH 6 (min)
mAb A	IgG1	0.396	9.1	2.85	3.09
mAb B	IgG1	0.368	9.0	3.03	3.27
mAb C	IgG1	0.396	9.3	2.25	2.77
mAb D	IgG2	0.406	7.8	3.32	3.94
mAb E	IgG1	0.507	8.0	3.80	5.16
mAb F	IgG1	0.386	8.2	3.51	4.47
mAb G	IgG2	0.452	7.3	4.98	7.63
mAb H	IgG1	0.433	9.1	N/A	N/A

Although the vendor and others (Beyer and Jungbauer 2018; Ladiwala et al. 2006; Ghose et al. 2013) have reported that TOYOPearl Butyl was more hydrophobic than TOYOPearl Phenyl, we found that TOYOPearl Phenyl was slightly more hydrophobic than TOYOPearl Butyl (Additional file 3: Table S1). One study had observed a greater change in T_m_ when comparing a mAb (CH14.18) adsorbing to TOYOPearl Butyl, to the same mAb adsorbing to TOYOPearl Phenyl (Beyer and Jungbauer 2018) which was consistent with an increased hydrophobicity for TOYOPearl Butyl. In that study, the relative hydrophobicity of TOYOPearl Butyl to that of TOYOPearl Phenyl appears to change as a function of ammonium sulfate concentration. The association constant K_a_ for CH14.198 decreased from 2.0 to 0.3 mg/mL for TOYOPearl Butyl and 0.3 to 0.2 mg/mL for TOYOPearl Phenyl when decreasing the ammonium sulfate concentration from 800 to 400 mM. It is possible that hydrophobicity of TOYOPearl Butyl is more sensitive to ammonium sulfate concentration than the hydrophobicity of TOYOPearl Phenyl and decreasing the ammonium sulfate to concentrations used in this study (350 mM) might decrease the hydrophobicity of TOYOPearl Butyl below that of TOYOPearl Phenyl. Additionally, Ladiwala et al. reported several proteins having longer retention times on a TOYOPearl Phenyl 650 M column compared to a TOYOPearl Butyl 650 M column using the same buffer condition (Ladiwala et al. 2006). These findings highlight the potential sensitivity of reported column hydrophobicity to both the buffer conditions and the specific molecules used for measurement.

### Relative surface hydrophobicity measurements

**Fig. 1:**
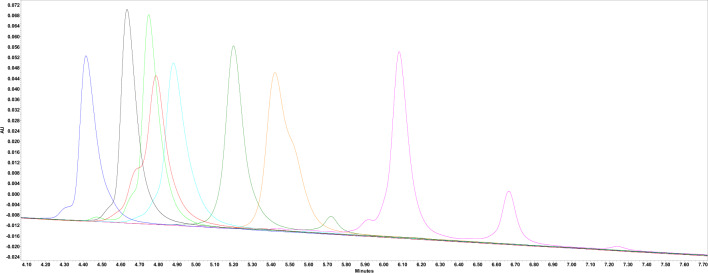
RP-HPLC chromatograms of each of mAbs using a TSK Butyl-NPR column with a decreasing ammonium sulfate concentration and increasing isopropanol concentration

The hydrophobicity of each molecule was qualitatively assessed by measuring its elution through a Butyl-NPR HPLC resin using an ammonium sulfate and isopropanol gradient. The chromatograms for each molecule are shown in Fig. 1. MAb B was the least hydrophobic and mAb E was the most hydrophobic. MAb B and mAb E share the same Fc region and, therefore, the differences between their apparent hydrophobicities are a result of differences in their Fab region. The RSH values for each mAb are listed in Table 2.

### ***Capillary zone electrophoresis***

CZE has previously been used to evaluate surface charge of proteins (Winzor et al. 2004). The surface charge of a protein is buffer dependent and can influence its chromatography behavior on HIC resins (Kårsnäs and Lindblom 1992). Therefore, the elution behavior of the mAbs in this study were measured under two different buffer conditions (400 mM EACA, 0.05% HPMC, pH 5 and 200 mM EACA, 0.05% HPMC, pH 6) to understand how pH can influence surface charge of these mAbs. It was necessary to adjust the concentration of EACA in each buffer to allow for usable currents in the CZE experiments. In each buffer condition, elution order of each of the mAbs corresponded with predicted order based on the pI of the mAb (Table 2).

### ***T***_***m***_*** and T***_***agg***_*** measurements***

Proteins can unfold in response to both heat and adsorption to a resin surface. T_m_ measures the thermal stability of a mAb as it unfolds in response to heat. As the mAb unfolds, internal hydrophobic patches become exposed leading to self-association and aggregation. MAbs can also unfold in response to the adsorption to a hydrophobic surface which can then lead to aggregation. T_m_ and T_agg_ were therefore used to draw a correlation between propensity of a mAb to unfold in response to heat and the propensity to unfold in response to contact with a hydrophobic surface. While thermal stress and adsorption to hydrophobic surfaces induce different changes in secondary structure (Vermeer et al. 1998) and therefore might induce different aggregate species, there may be some mutual underlying relation between the two that allows for correlative studies (e.g., domain stability).

**Fig. 2:**
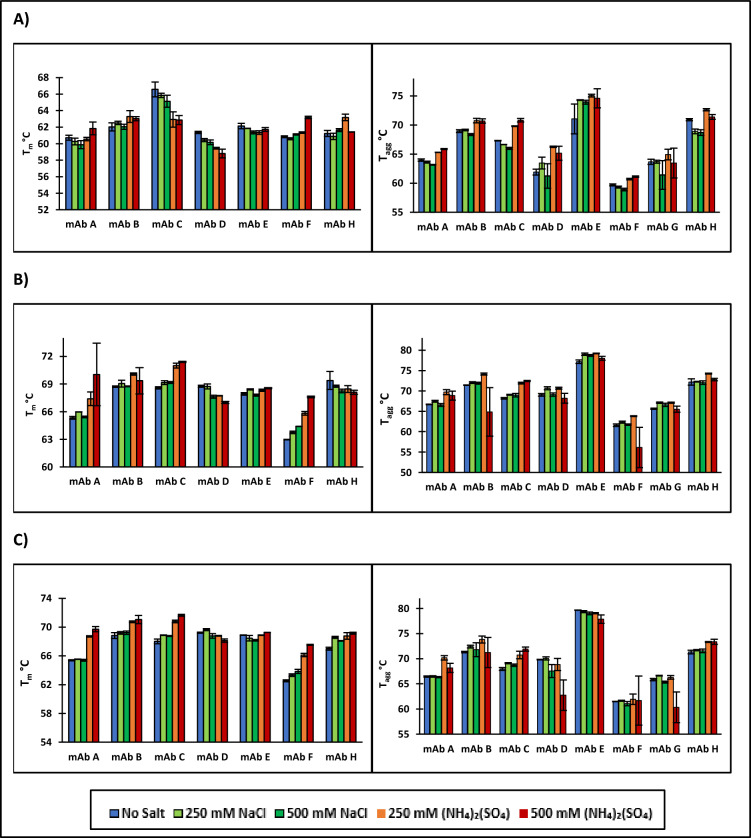
T_m_ (left) and T_agg_ (right) measurement for each of the mAbs used in this study in 50 mM sodium citrate **A** pH 5, **B** pH, 6, and **C** pH 7. The T_m_ of mAb G could not be determined using our DSF method

The thermal unfolding of mAbs occurs across multiple transitions corresponding to the unfolding of the CH2, Fab, and CH3 domains (Majumdar et al. 2013; Brader et al. 2015; Garber and Demarest 2007; Franey et al. 2010). However, using our DSF methods (see Methods section), there was only one reproducibly observable T_m_ seen across the screened mAbs in our study. The inability to consistently distinguish multiple T_m_ values could be a result of the lower signal-to-noise ratio of the fluorescence peak intensity at higher temperatures. Another study using the fluorescence emission ratio at 350/330 nm was also only able to observe one T_m_ but was able to observe three transitions for the same mAbs when using DSC (Brader et al. 2015). The T_m_ and T_agg_ values for the first observed melting point are summarized in Fig. 2. The first T_m_ is typically associated with the unfolding of the CH2 domains (Tischenko et al. 1998), though this was not determined for the mAbs in this study. MAb G did not have an observable T_m_. The BCM for the native form of mAb G occurs at a longer wavelength than any other mAb used in this study and near the BCM for each of the unfolded mAbs (Table 3). MAb G also exhibits only a minor change in BCM upon unfolding suggesting that the tryptophan residues in mAb G are already solvent exposed. However, since T_agg_ depends on SLS at 266 nm, T_agg_ measurements for mAb G could be measured.

**Table 3:** BCM of the fluorescence emission spectrum at 25 °C and 75 °C in 50 mM sodium citrate pH 5 for each of the mAbs used in the study

mAb	BCM @ 25 °C (nm)	BCM @ 75 °C (nm)	Difference (nm)
mAb A	346.48	348.64	2.15
mAb B	344.78	349.40	4.62
mAb C	343.42	349.60	6.18
mAb D	346.85	348.41	1.56
mAb E	345.69	347.98	2.29
mAb F	341.14	346.12	4.98
mAb G	347.51	348.03	0.52

MAb A, mAb D, and mAb F tended to have the lowest T_m_ and T_agg_ values across almost all measured buffer conditions. Despite having the same Fc region, mAb B and mAb D had different T_m_ values. Since the first T_m_ is generally associated with unfolding of the CH2 region, the difference could arise because of interactions between the F_ab_ and the F_c_ domains if the observed T_m_ corresponds to the unfolding of the CH2 region. For T_agg_ measurements, mAb B, mAb C, and mAb E had the highest T_agg_ and are expected to be the most stable.

All mAbs exhibited an > 2 °C increase in T_m_ (except mAb G) and T_agg_ when increasing from pH 5 to pH 6 and smaller increase in T_m_ from pH 6 to pH 7. Given the pH range in which the increase in T_m_ occurs, the increase in T_m_ is likely a result of a change in protonation state of a histidine residue (pKa ~ 6). Additionally, because the first measured T_m_ usually corresponds to the unfolding of the CH2 domain, the histidine is likely in the CH2 domain of these mAbs. Ionescu et al. also observed an increase in T_m_ of the CH2 domain when increasing the pH from 5.5 to 6.5 on two different mAbs (Ionescu et al. 2008). This stabilization of the CH2 seems linked to resistance to thermal unfolding and potentially subsequent aggregation.

### HTP studies

**Fig. 3:**
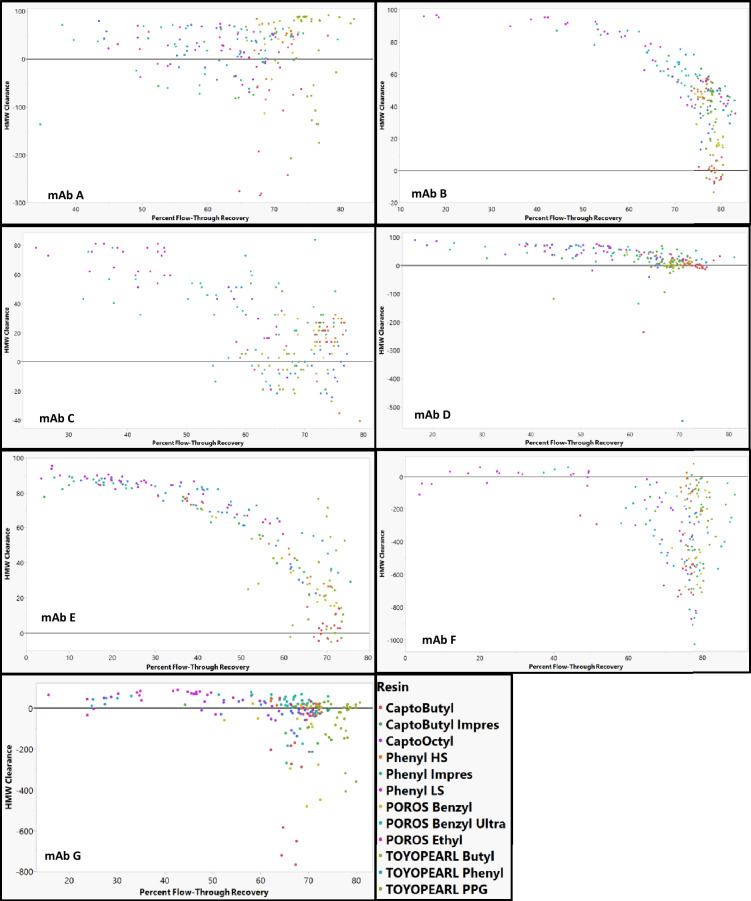
Percent flow-through recovery vs HMW clearance for each of the mAbs in the study across different HIC resins

Percent flow-through recovery vs HMW clearance plots for each of the mAbs are shown in Fig. 3. Across each of the mAbs in this study, an increase in percent flow-through recovery generally resulted in a decrease HMW clearance. However, this trend was not as strong for mAb A, mAb F, and mAb G. These mAbs also had negative HMW clearance (i.e., generation of HMW species) under several resin–buffer conditions. This generation of HMW species might explain why the trend was not observed for these mAbs. MAb A, mAb F, and mAb G were the most prone to generating HMW species which is consistent with the low stability of these mAbs (Fig. 2). For mAb A, the dimer was the dominant HMW species in both the load material and in the material where HMW species was generated. In contrast, while the dominant HMW species in mAb F load material was a dimer, the flow-through consisted of species consistent with higher order aggregates ranging from trimers to decamers. Similarly, the main HMW species in the mAb G load material was a dimer, but a tetramer was the dominant HMW species in the flow-through material in which HMW species was generated. Interestingly, for mAb G, ButylS FF and Phenyl FF LS (two of the least hydrophobic resins) induced large amounts of HMW species. Lower hydrophobic resins are generally thought to induce fewer conformational changes than higher hydrophobic resins. Therefore, it is surprising that these low hydrophobicity resins resulted in such large amounts of aggregation.

The HMW species observed across mAb A, mAb F, and mAb G are an irreversible form of aggregation and not a buffer-dependent self-association. In our SEC analysis methods, the sample volume is < 0.5% of the column volume. As the sample enters the column, the sample is buffer exchanged into the mobile phase buffer. Therefore, the HMW species generated were not a reversible self-association of the mAbs, but a result of irreversible aggregation as a result of conformational changes.

### Modeling

Because of the complexity of the data set, percent flow-through recovery and HMW clearance were modeled for each individual resin and mAb to make the identification of individual point outlier and unique mAb–resin interactions easier. Additionally, percent flow-through recovery and HMW clearance were modeled individually to further simplify the models. All models use pH, salt type, and salt concentration as descriptors in addition to descriptors for either the mAb or the resin as discussed in the text. Further details of generating the models are in the Methods section.

### Modeling percent recovery on individual resins

**Fig. 4:**
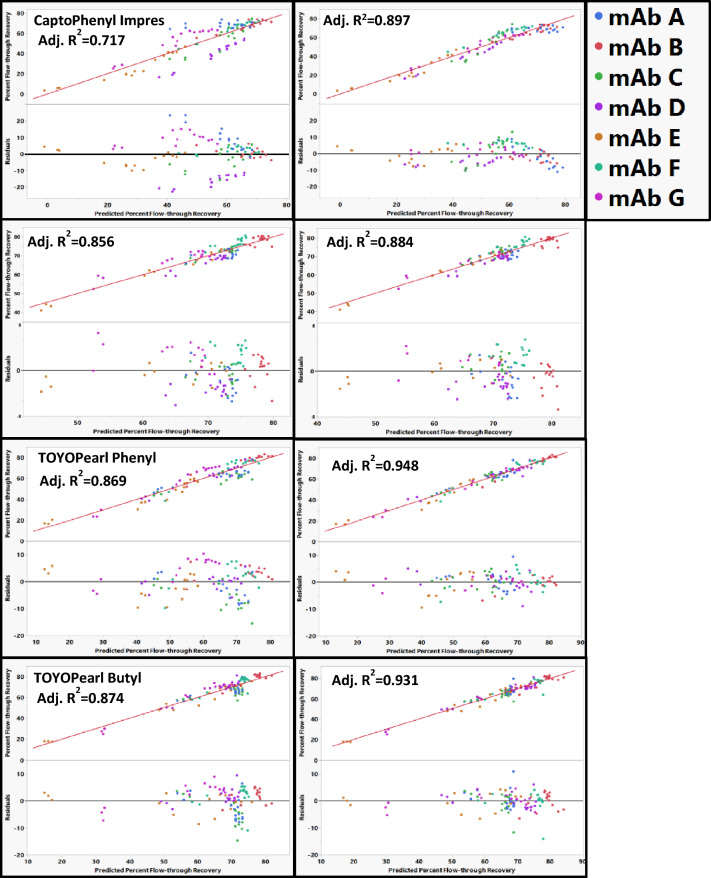
Actual vs predicted plots for percent flow-through recovery on CaptoPhenyl ImPres, Phenyl FF LS, TOYOPEARL Phenyl and TOYOPEARL Butyl resin. Residual plots shown directly beneath the actual vs predicted plots using (left) RSH alone as mAb main effects and (right) RSH and pI as mAb main effects. The identity line is shown in red as a reference

RSH was initially used as a single main parameter to describe the mAbs in the models as the hydrophobic interaction is the dominant mAbs resin interaction. Capto Phenyl ImPres, Phenyl FF LS, TOYOPearl Butyl, and TOYOPearl Phenyl could all be adequately fit with using just RSH without any observable mAb outliers (Fig. 4). These resins were then re-fit using RSH and either pI or the elution time in the CZE assays to describe both the surface hydrophobicity and charge of the mAbs. When resins were modeled with RSH and pI, the models resulted in higher adjusted *R*^2^ and with no observable mAb outliers (Fig. 4). Using RSH and pI as mAbs descriptor gave excellent fits for the prediction of percent flow-through recovery for these resins.

**Fig. 5:**
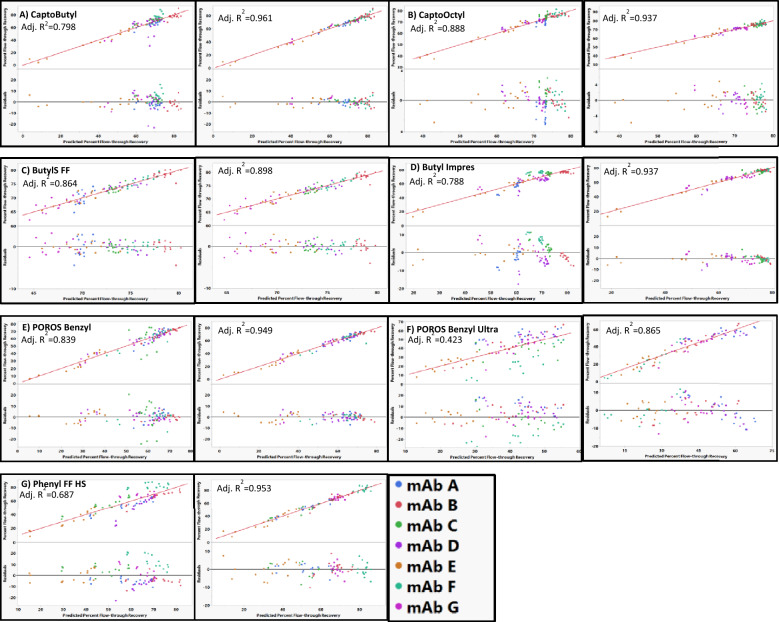
Actual vs predicted plots for resin models using RSH and pI for all mAb (left) and removed mAb outlier (right) for **A** CaptoButyl, **B** CaptoOctyl, **C** Butyl S FF, **D** Butyl ImPres, **E** POROS Benzyl, **F** POROS Benzyl Ultra, and **G** Phenyl FF HS. MAb D was removed from CaptoButyl and Phenyl FF HS. MAb A was removed from CaptoOctyl, ButylS FF, and Butyl ImPres. MAb C was removed from POROS Benzyl and POROS Benzyl Ultra. The identity line is shown in red as a reference

The best fit models for Capto Butyl, Capto Octyl, ButylS FF, and Capto Butyl ImPres, all had unique mAb–resin interactions when fitting the percent flow-through recovery using RSH and pI as mAb descriptors (Fig. 5). For mAb D on Capto Butyl, the model predicted approximately the same percent flow-through recovery values (65–70%) across a wide range of actual percent recovery values resulting in systematic skewing of residuals at lower percent flow-through recovery values. Removing mAb D from the Capto Butyl model resulted in improved adjusted *R*^2^ and no discernable outliers (Fig. 5). We observed similar behavior for mAb A on Capto Butyl ImPres, ButylS FF, and CaptoOctyl (Fig. 5). Predicted values for mAb A systematically deviated from the actual values on these resins compared to the other mAbs. It is unclear why these mAbs exhibit different retention behavior. MAb A does not have any distinguishing characteristics based on properties measured in this study. While mAb D appears as an outlier on Capto Butyl, mAb D was adequately modeled on Capto Butyl ImPres. Similarly, mAb A was adequately modeled on Capto Butyl, but not on Capto Butyl ImPres. Particle size is the main difference between Capto Butyl (75 μm) and Capto Butyl ImPres (40 μm) (Additional file 3: Table S1) and it has been shown that particle size influences peak resolution by decreasing mass transfer resistance (Kimerer et al. 2020). However, given that the behavior of mAb A fit well when modeling CaptoPhenyl ImPres (particle size 40 μm), the unique behavior of mAb A on Capto Butyl ImPres may not solely be a result of particle size. Additionally, mAb A appeared as an outlier when modeling ButylS FF and CaptoOctyl resin which both have a particle size of 90 μm (Additional file 3: Table S1). Therefore, there might be some other attributes of the resins that cause these unique interactions with these mAbs.

POROS Benzyl, POROS Benzyl Ultra, and Phenyl FF HS also had unique mAb outliers when modeling using RSH and pI as descriptors for the mAbs (Fig. 5). For the POROS Benzyl model, mAb C had poor residual distribution (Fig. 5). As mentioned in discussion of resin hydrophobicity measurements, mAb C had an unexpectedly lower retention on POROS Benzyl compared to other aromatic ligands. Removing mAb C from the POROS Benzyl model resulted in an improved adjusted *R*^2^ (Fig. 5). The inability to fit mAb C using this model may be related to the unique behavior on POROS Benzyl discussed in resin hydrophobicity measurement section. The model for POROS Benzyl Ultra had poor adjusted *R*^2^ and no distinct mAb outlier in terms of residual distribution. However, since mAb C was an outlier on POROS Benzyl and because the POROS Benzyl and POROS Benzyl Ultra resins differ only in ligand density, mAb C was excluded from the POROS Benzyl Ultra model. Excluding mAb C from the model resulted in a substantial increase in adjusted *R*^2^ (Fig. 5). Phenyl FF HS also could not be adequately modeled when including all mAbs in the initial model with mAb D deviating the most from the model and negative residual values for all buffer conditions (Fig. 5). We currently have insufficient structural information to develop a mechanistic evaluation to explain this behavior. Removing mAb D from the model resulted in improved adjusted *R*^2^ and residual distribution (Fig. 5).

**Fig. 6:**
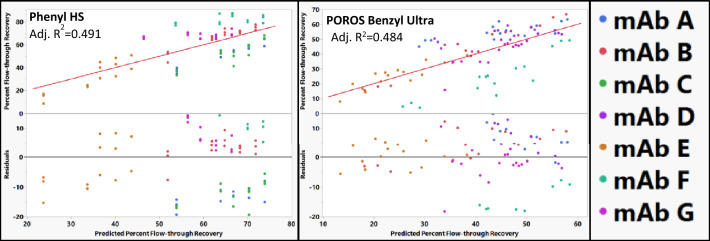
Actual vs predicted percent flow-through recovery plots and residual plots for Phenyl HS and POROS Benzyl Ultra using only RSH as a mAb descriptor. RSH alone was not able to adequately model flow-through behavior for these resins. The identity line is shown in red as a reference. No outliers were removed based on residual size in order to demonstrate poor residual distributions

In contrast to the previously discussed resins, both POROS Benzyl Ultra and Phenyl FF HS had poor quality of fits (*R*^2^ ~ 0.5) when using RSH alone to describe the mAb even when mAb C and mAb D were excluded (Fig. 6). The substantial increase in the quality of fit when including the pI compared to the other resins could be because the retention mechanism on these resins involves surface charge interactions. Both these resins are highly substituted aromatic ligands. Therefore, including surface charge might account for cation–π interaction between the mAb and the aromatic ligands of the resins.

**Fig. 7:**
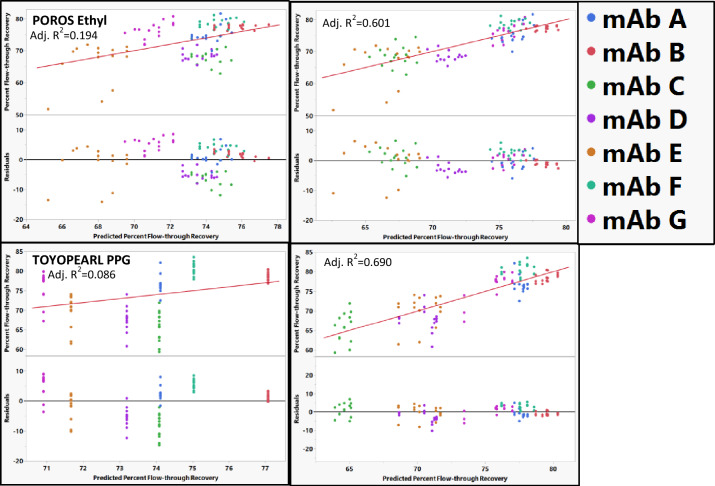
Actual vs predicted and residual plots for POROS Ethyl and TOYOPearl PPG which could not be fit using RSH (left) or RSH and pI (right). The identity line is shown in red as a reference

POROS Ethyl and TOYOPearl PPG could not be modeled with RSH and pI as descriptors (Fig. 7). It is possible that POROS Ethyl has a different retention mechanism compared to the other resins. POROS Ethyl resin is synthesized by coupling a cross linkable monomer containing an ethyl group to the Poly(styrene-DVB) support. The reason we could not model the percent recovery for POROS ethyl could be because of interactions between the mAb and the functional unit used to couple the ethyl group to the resin. TOYOPearl PPG contains a terminal hydroxyl group in the polypropylene glycol ligand. The hydroxyl group can have hydrogen bond interactions which might not be fully accounted for in these models. Therefore, the unique structural properties of these resins might prevent them from accurately being modeled using our methods.

Using RSH and CZE elution times gave comparable or slightly better quality of fits compared to RSH and pI (Additional file 2: Fig. S2). We observed similar mAb outliers when modeling using RSH and CZE elution time as we did when modeling using RSH and pI. Since pI measurements are a more routine measurement within industry, the pI was chosen over the CZE elution time as a molecular descriptor for surface charge. All further models included RSH and pI as mAb descriptors.

### Modeling percent recovery for individual mAbs

**Fig. 8:**
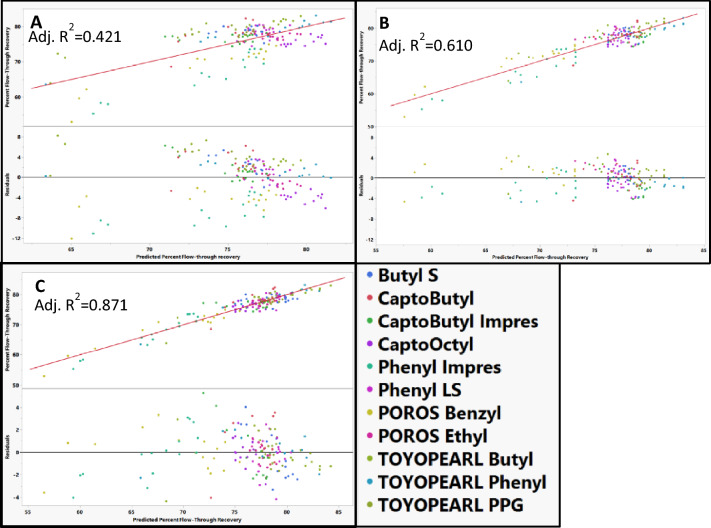
Actual vs predicted percent flow-through recovery plots and residual plots for mAb B across different resins using **A** resin hydrophobicity, **B** resin hydrophobicity and zeta-potential, **C** resin hydrophobicity, zeta-potential, and vendor as resin descriptors for the model. The identity line is shown in red as a reference

Resin hydrophobicity, resin zeta-potential, and resin vendor were evaluated as model effects to describe the resin when modeling percent flow-through recovery for individual mAbs across the different resins. Using mAb B as a representative example mAb, resin hydrophobicity alone could not model percent flow-through recovery (Fig. 8). Including streaming zeta-potential measurement resulted in a large increase in adjusted *R*^2^ (0.421 vs 0.610). The precise explanation for the enhanced quality of fit resulting from the inclusion of zeta-potential in the model is still not entirely known. The hydrophobic resins do not contain charged functional groups. The zeta-potential may arise because of the adsorption of ions (hydroxide, hydronium, potassium, and chloride ions) to the surface of HIC resin. The adsorption of these ions to uncharged hydrophobic surfaces has been reported previously (Stubenrauch et al. 2002; Hozumi et al. 2001; Zimmermann et al. 2010) and further studied computationally (Kudin and Car 2008; Zangi and Engberts 2005). Because the zeta-potential reflects the extent of adsorbed ions, the zeta-potential measurement could mechanistically relate to the hydrophobic interaction in several ways (i) the adsorbed ions would have to desorb from the resin surface prior to adsorption of the mAb to the resin; (ii) the adsorbed ions could also affect the hydrophobic interaction by altering the structure of water molecules on the resin surface) the zeta-potential may reflect morphological features of the resin affecting hydrodynamic flow of ions through the resin similar to what has been observed in hydrophobic polymers (Zimmermann et al. 2010). The zeta-potential measurements of the resins were performed using a different buffer (10 mM KCl) than what was used in the HTP studies. Therefore, the effect of additional buffer components on the zeta-potential measurements and surface properties of the resin are not considered. The charge profile of HIC resins is generally not considered when modeling HIC resin chromatography behavior. To our knowledge, this observation is one of the first reports to explore the significance of the resin surface charge in HIC resins. There are various contributors to the zeta-potential and more studies should be performed to understand the significance of zeta-potential on the hydrophobic interaction. Lastly, including the vendor as a main effect to account for differences in the support of the different resins further increases the adjusted *R*^2^ to 0.876. Other descriptors of the resin such as particle size and porous structure were not considered because of the collinearity with the vendor descriptor. Additionally, hydrophobicity, zeta-potential, and vendor could adequately model percent recovery and therefore further descriptors were not used. Resin hydrophobicity, zeta-potential, and vendor were used to model the percent recovery for the rest of the mAbs used in the study.

**Fig. 9:**
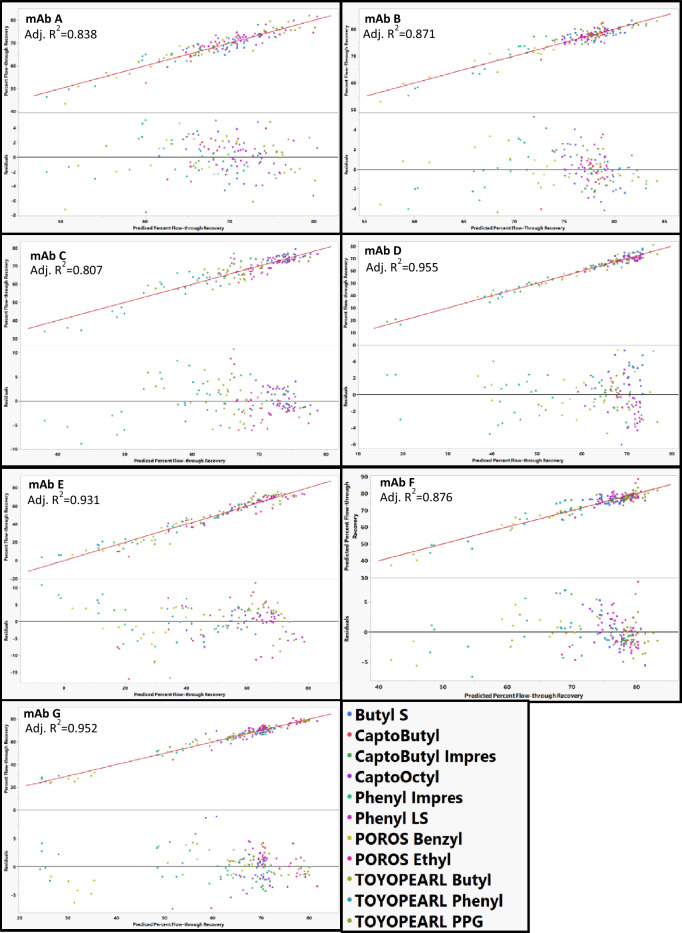
Best models actual vs predicted percent flow-through recovery plots and residual plots for mAb in the study across different resins. POROS Benzyl was removed from MAb C, CaptoButyl was removed from MAb D, and ButylS FF was removed from MAb E. The identity line is shown in red as a reference

**Fig. 10:**
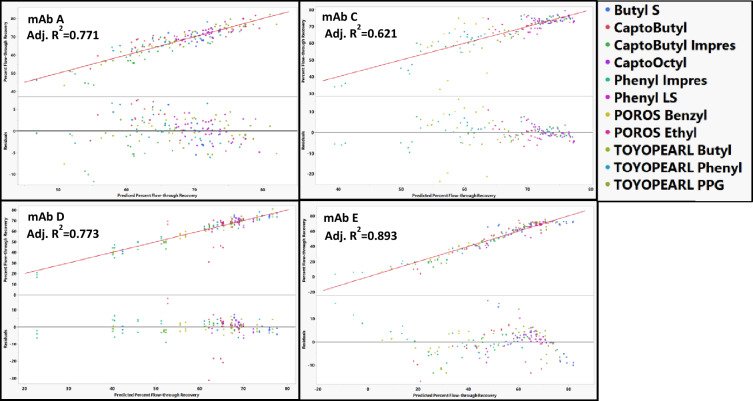
Actual vs predicted percent flow-through recovery and residual plots for mAb A, mAb C, mAb D, and mAb E with all resins included in the model. CaptoButyl ImPres was an outlier on mAb A, POROS Benzyl was an outlier for mAb C, CaptoButyl was an outlier for mAb D, and ButylS was an outlier for mAb E. The identity line is shown in red as a reference. No outliers were removed based on residual size in order to demonstrate poor residual distributions

The best fit models for each of the mAbs are shown in Fig. 9. mAb B, F, and G could be modeled using resin hydrophobicity, zeta-potential, and vendor as main resin descriptors without observable resin outliers. However, Capto Butyl ImPres, POROS Benzyl, Capto Butyl, and ButylS FF appeared as outliers for mAb A, mAb C, mAb D, and mAb E, respectively (Fig. 10). Removal of these resins from the individual model led to adequate fits for the models. When modeling percent recovery on individual resins, the same mAb A appeared as an outlier when modeling Capto Butyl ImPres, as did mAb C when modeling POROS Benzyl, and mAb D when modeling Capto Butyl. In contrast, while the best fit model of ButylS FF adequately modeled the percent flow-through recovery for mAb E, the best model for mAb E was unable to predict the percent flow-through recovery for ButylS FF. The reasons why the model for mAb E was unable to account for ButylS FF are unclear. The model can adequately predict the behavior of Capto Butyl, Capto Butyl ImPres, and TOYOPEARL Butyl. A major difference between these resins and ButylS FF resin is that a sulfur atom connects the butyl ligand to resin support as opposed to an oxygen atom in the other resins. The S-ether might cause unique interaction with mAb E that we are not able to assess using the descriptors in our models.

### Modeling HMW clearance for individual mAbs

**Fig. 11:**
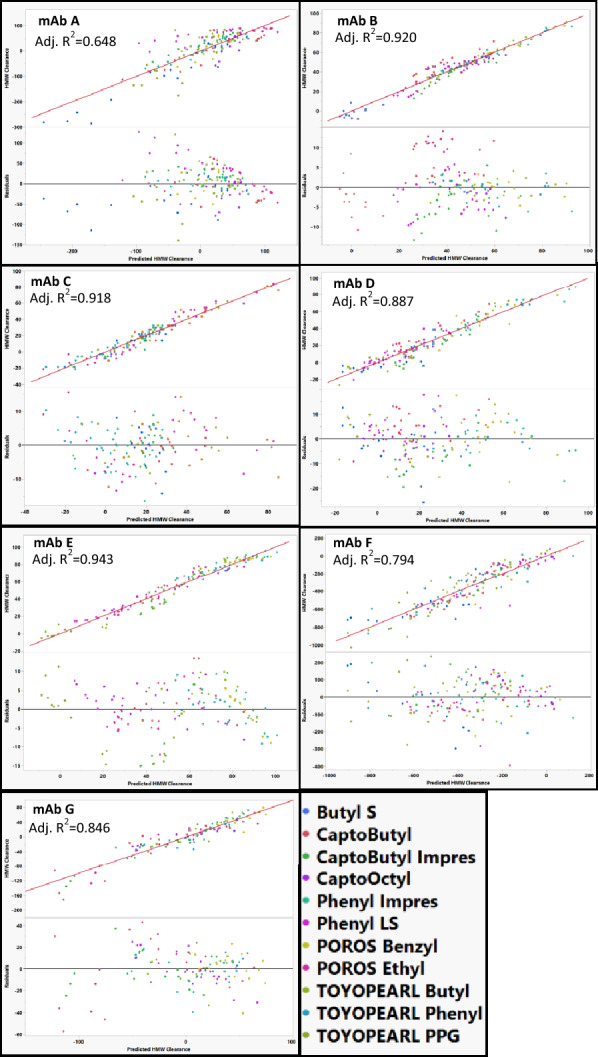
Best actual vs predicted and residual plots for models predicting HMW clearance for each mAb. The identity line is shown in red as a reference. TOYOPEARL PPG was removed when fitting mAb A, Butyl ImPres was removed when modeling mAb C, ButylS FF was removed when modeling MAb E, Butyl S, Phenyl FF LS, and TOYOPEARL PPG were removed from mAb G

All models for HMW clearance used the same descriptors as the ones used to model percent flow-through recovery. When using these descriptors, mAb B and mAb D could be modeled without apparent outlying resins (Fig. 11). Interestingly, in contrast to the percent flow-through recovery models, Capto Butyl was not an outlier when modeling mAb D and neither was POROS Benzyl an outlier on mAb C. It is likely that the mechanisms involved in percent flow-through recovery are different than the mechanisms involved in HMW clearance.

**Fig. 12:**
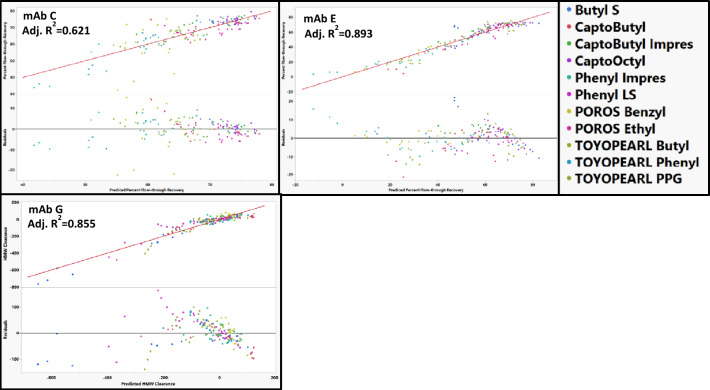
Actual vs predicted HMW clearance and residual plots for mAb C, mAb E, and mAb G with all resin included in the model. The identity line is shown in red as a reference. Capto Butyl ImPres appeared as an outlier for mAb C, Butyl S FF appeared as an outlier for mAb E, and Butyl S FF, POROS Ethyl, and Phenyl FF LS appeared as outlier for mAb G. The identity line is shown in red as a reference. No outliers were removed based on residual size in order to demonstrate poor residual distributions

HMW clearance could not be properly modeled for mAb C, mAb E, and mAb G without removing data points for specific resins. Including all resins in the model for mAb C, resulted in the model overestimating the actual HMW clearance for almost all buffer conditions on Capto Butyl ImPres. Therefore, Capto Butyl ImPres was removed from the model for mAb C. The model for mAb E was not able to accurately predict the HMW clearance behavior for ButylS FF resulting in a poor residual distribution (Fig. 12). Removal of ButylS FF from the model resulted in improved quality of fit for the model (Fig. 11). For mAb G, there was biphasic behavior in the actual vs predicted plots (Fig. 12) with an inflection around − 200% HMW Clearance. TOYOPEARL PPG, Phenyl FF LS, and Butyl S FF comprise the region below − 200% HMW clearance indicating formation of HMW species. For Phenyl FF LS and TOYOPEARL PPG, the HMW Clearance below –200% occurs at pH 7 and for ButylS FF the points in the low HMW Clearance region occur at pH of 6 and 7. MAb G is the most acidic mAb with a pI of 7.3 (Table 2) and the decrease in HMW clearance might be a result of decreased stability on the resin near its pI. This biphasic behavior for mAb G might be caused by two different mechanisms related to the HMW clearance behavior: one mechanism relates to the HMW clearance, and another relates to the HMW generation. Both can occur simultaneously, but at low HMW clearance the mechanism for HMW generation dominates and at higher HMW clearance the mechanism for HMW clearance dominates. Because of this finding, TOYOPEARL PPG, Phenyl FF LS and Butyl S FF were removed from the model for mAb G.

Models for mAb A and mAb F poorly predicted HMW clearance for multiple resins (Fig. 11). The model for mAb A resulted in poor quality of fit with poor residual distribution across several resins. The residuals for mAb A were often large (> 50%) and included a poor non-random distribution. Several conditions lead to negative HMW clearance values for mAb A which might contribute to the poor model quality. Likewise, for mAb F, the descriptors we used resulted in a poor fit for our model and large residuals (> 100% HMW clearance). The poor fits for mAb F might be a result of its poor stability (Fig. 2) leading to large amount of HMW generation in our study. The descriptors in our models may not be able to capture the features of the resin associated with HMW species generation for mAb F.

### Modeling HMW clearance for individual resins

HMW clearance for each individual resin could not be modeled when either mAb A or mAb F were included. Because the amount of HMW species generally increased on these resins (Fig. 2), models were unable to account for conditions of both HMW generation and HMW clearance. Therefore, mAb A and mAb F were excluded from all resin models.

**Fig. 13:**
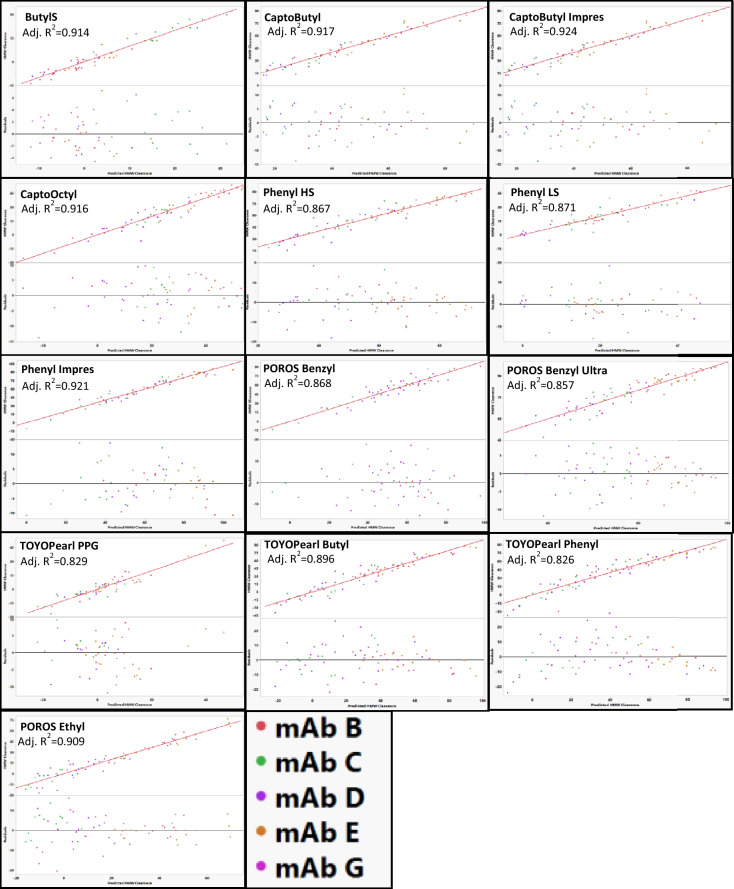
Actual vs predicted and residual plots for best fit resin models. MAb E was removed from POROS Benzyl. MAb G was removed from ButylS, CaptoPhenyl LS, TOYOPEARL PPG, Phenyl ImPres, and Capto Butyl. Actual vs predicted and residual plots for POROS Benzyl, ButylS FF, Phenyl FF LS, CaptoOctyl, and TOYOPEARL PPG with all mAbs are in the supporting information. The identity line is shown in red as a reference

**Fig. 14:**
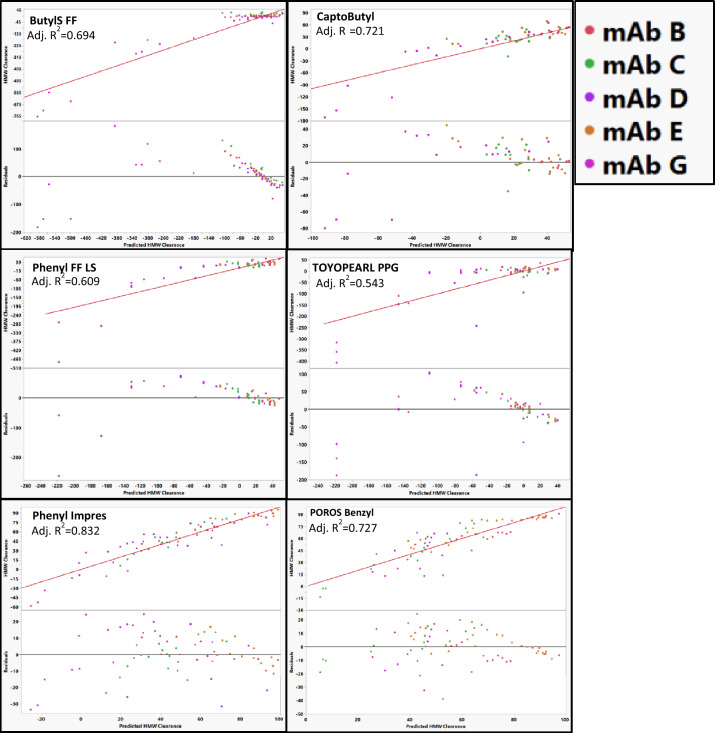
Actual vs predicted HMW clearance plots and residual plots for ButylS, Capto Butyl, Capto Phenyl LS, TOYOPEARL PPG, Phenyl ImPres, and POROS Benzyl with all mAbs included except mAb A and mAb F. MAb G appeared as an outlier on ButylS, Capto Butyl, Phenyl FF LS, TOYOPearl PPG, and Phenyl ImPres and mAb E appeared as an outlier on POROS Benzyl. The identity line is shown in red as a reference. No outliers were removed based on residual size in order to demonstrate poor residual distributions

When modeling HMW clearance by resins, POROS Ethyl, POROS Benzyl Ultra, Phenyl FF HS, TOYOPearl Butyl, TOYOPearl Phenyl, CaptoOctyl, and Capto Butyl ImPres could all be modeled just using RSH and pI as mAb descriptors (Fig. 13). MAb G was an outlier on ButylS FF, Phenyl FF LS, TOYOPearl PPG, Phenyl ImPres, and Capto Butyl (Fig. 14). The low stability of mAb G (Fig. 2) and conditions that led to increases in HMW species (Fig. 3) might contribute to the difficulty in modeling mAb G. The outlying points are of low and sometimes negative HMW clearance in each of the resins where a mechanism related to HMW generation is expected to dominate. When modeling HMW clearance for POROS Benzyl, predicted HMW clearance values for mAb E resulted in poor residual distribution (Fig. 14). Therefore, mAb E was removed from the model for POROS Benzyl.

### Application of models to column chromatography selection

To demonstrate how these models could be used to guide the selection of a HIC column, the models were applied to a test mAb (mAb H). Our model predictions are for percent recovery and HMW clearance based on a batch-binding configuration. The percent recovery values in the HTP technique may not translate to percent recovery values in a column format because of differences in collection criteria between the two methods. In a column format, the flow-through material is typically collected based on peak collection criteria in which buffer is flown over the column to elute out the entire portion of a peak the meets a specified absorbance threshold. Low percent recoveries in the HTP format would lead to greater volume of buffer required for elution of the mAb. Therefore, high percent recoveries in the HTP format would still lead to favorable outcomes in the column format although they might not be reflective of actual percent recoveries in a column format. Ideally, the optimal resin would have both high predicted HMW clearance and percent recovery values. Additionally, the test mAb should have a high T_m_ and T_agg_ values to avoid generation of HMW species because of interaction with the HIC resin. By using thermal stability measurements and predicted percent flow-through recovery and HMW clearance, we could assess the suitability of HIC for purifying mAb H and guide the selection for an appropriate column for its purification.

**Fig. 15:**
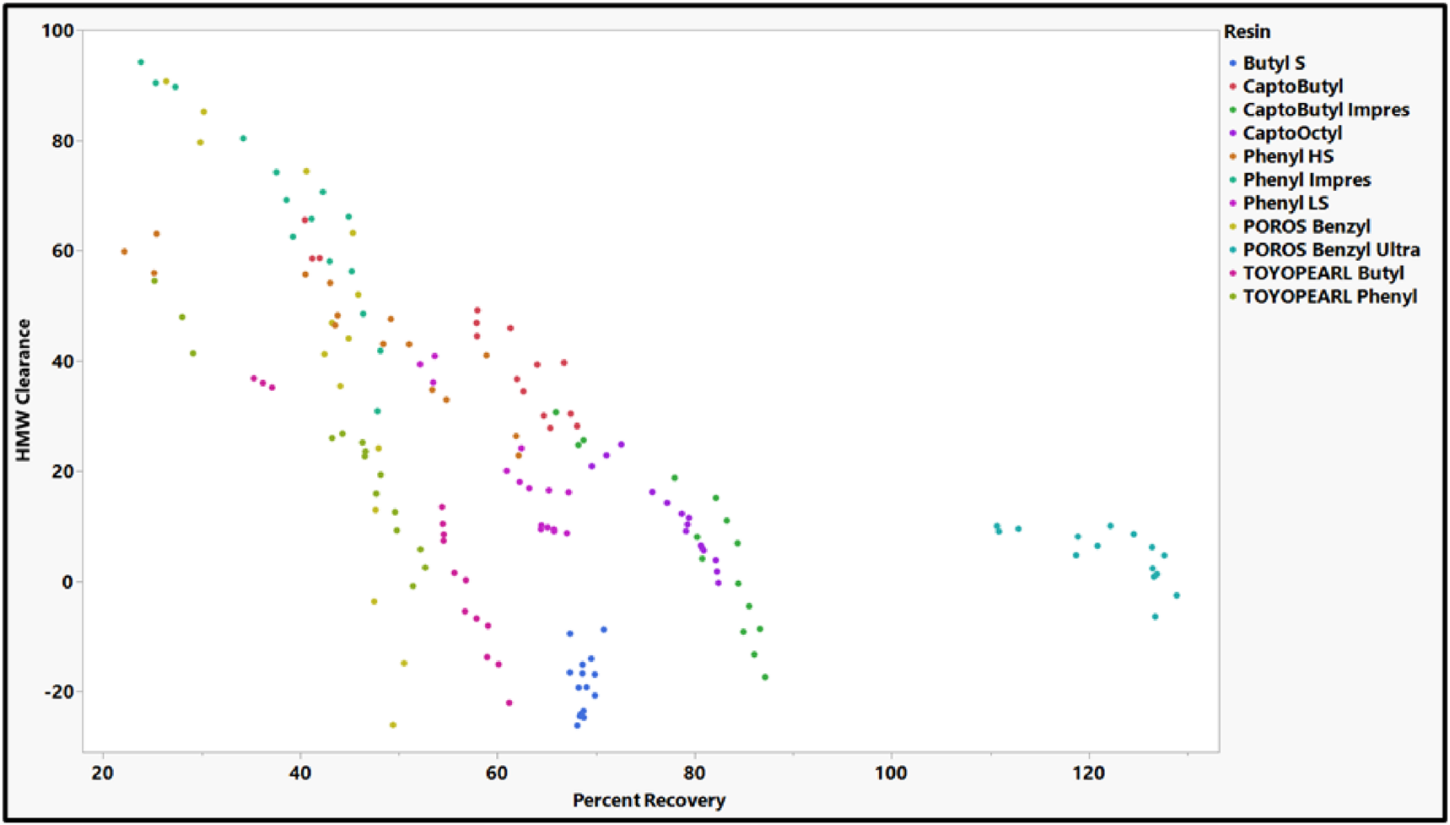
Predicted HMW clearance vs percent flow-through recovery plot for mAb H based on individual resin models

**Fig. 16:**
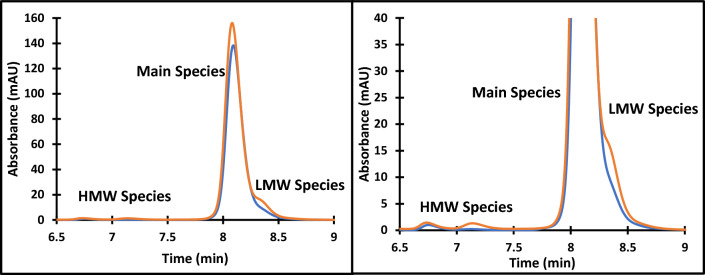
SEC chromatogram of mAb H CaptoButyl load material (orange) and CaptoButyl eluate (blue) (left) and a zoomed in view of the HMW and LMW region (right). Absorbance was measured at 280 nm

The thermal stability and the modeling studies were used to develop a process for HIC resin selection for HMW clearance using a test mAb (mAb H). First, T_m_ and T_agg_ should be assessed to determine suitability for HIC chromatography. For mAbs with low T_m_ (~ 60 °C) and T_agg_ values (~ 65 °C), purification by HIC chromatography may not be the best option due to the potential for increase in HMW species. The high T_m_ and T_agg_ of mAb H (Fig. 2), suggest that it will be stable during the HIC process and would likely not lead to HMW generation on the column. Next, the individual resin models were used to predict optimal resin and buffer conditions in terms of both percent flow-through recovery and HMW clearance using the RSH (0.433) and pI (9.05) of mAb H. The predicted percent flow-through recovery and HMW clearance for mAb H for each resin are shown in Fig. 15. Potential optimal resins would lie on the outside front of plot in Fig. 15. Capto Butyl, Capto Butyl ImPres, POROS Benzyl, and Phenyl ImPres lie on this outside front of this plot. While Capto Butyl ImPres and POROS Benzyl have a high HWM clearance, they also occupy a region of low percent recovery (< 40%) making them unsuitable for purifying mAb H. Phenyl ImPres has a high percent recovery but a low HMW clearance leading to an expected low HMW clearance on a column chromatography format. In Fig. 15, predicted values for Capto Butyl appeared in a region of both high HMW clearance and high percent flow-through recovery. Therefore, Capto Butyl covers the expected design space that a typical downstream process development step would use. When mAb H was purified using a Capto Butyl resin with a 50 mM Sodium Citrate pH 5 buffer, there was an 89% recovery and a 60% HMW clearance (Fig. 16). In addition to removing the HMW species, the purification process decreased low molecular weight species. However, there was not enough resolution between the main and the LMW species in the eluate to quantify LMW clearance in the eluate. The high percent recovery and high HMW clearance obtained using a bench scale purification suggest that this predictive modeling approach could be used in future application for identifying HIC resins for the purification of other mAbs.

## Conclusion

The goal of this study was to develop predictive models as a selection tool for choosing HIC resins for the removal of HMW species. In this study, we used measurements of thermal stability, surface hydrophobicity, and surface charge to predict behavior of mAbs across several HIC resins. Our thermal stability studies suggest that low T_m_ and T_agg_ leads to greater generation of HMW species on resins. We were able to adequately model the percent flow-through recovery and HMW clearance of different mAbs on single HIC resin using just RSH and pI of a mAb. Similarly, we were able to model how a single mAb would behave across different HIC resins using measurements of hydrophobicity, zeta-potential, and vendor to describe the properties of the resin. In models for both the mAbs and the resins, outliers were identified and removed largely based on poor residual distribution. This study is one of the first reports to identify the significance of zeta-potential in the chromatography behavior of HIC resins. We successfully applied the models to guide the resin selection for high percent recovery and HMW clearance for a test mAb. This approach to modeling HIC resins performance could potentially be extended to predicting removal of other impurities in downstream processing such as HCP and DNA.

## Supplementary Information

**Additional file 1: Figure S1.** Chromatogram for the injection of 50 μL 20 mg/mL 150 kDa Dextran on a TOYOPearl Phenyl resin using a 25 mM Sodium Citrate, 5 % Isopropanol mobile phase. Dextran exhibits strong retention on TOYOPearl Phenyl resin under low salt and mild organic phase conditions. Absorbance was measured at 260 nm.

**Additional file 2: Figure S2.** Best Actual vs Predicted and Residual plots for models predicting HMW Clearance for each resin when using RSH and CZE elution time at pH 5 as mAb descriptors. The identity line is shown in red as a reference. MAb A was an outlier on CaptoButyl ImPres and Butyl S, mAb C was an outlier on POROS Benzyl and POROS Benzyl Ultra, mAb D was an outlier on CaptoButyl and Phenyl HS and were excluded from the model.

**Additional file 3: Table S1.** Properties of the HIC resins used in this study. Particle size is the average particle size as reported by the vendor.

## Data Availability

The datasets generated and/or analyzed during the current study are not publicly available due to legal and confidentiality concerns but are available from the corresponding author on reasonable request.

## Abbreviations

AEX: Anion exchange
BCM: Barycentric mean
CEX: Cation exchange
CHO: Chinese hamster ovary
CV: Column volume
CZE: Capillary zone electrophoresis
DOE: Design of experiment
HCP: Host cell protein
HIC: Hydrophobic interaction chromatography
HMW: High-molecular weight
HTP: High-throughput plate
HTS: High-throughput screening
mAb: Monoclonal antibody
MMC: Multi-modal chromatography
SLS: Static light scattering
T_agg_: Aggregation temperature
T_m_: Melting temperature
