# Correction: Modeling the behavior of monoclonal antibodies on hydrophobic interaction chromatography resins

**DOI:** 10.1186/s40643-026-01089-2

**Published:** 2026-06-26

**Authors:** Douglas Nolan, Thomas R. Chin, Mick Eamsureya, Sheldon Oppenheim, Olga Paley, Christina Alves, George Parks

**Affiliations:** 1 Takeda Pharmaceuticals America Inc, Lexington, MA 02421 USA; 2 Eurofins Lancaster Laboratories Professional Scientific Services, LLC, Lancaster, PA 17601 USA

**Correction: Bioresources and Bioprocessing (2024) 11:25** 10.1186/s40643-024-00738-8

In the article (Nolan et al. 2024) the authors have identified an error in Table S1 and the caption of Figure 16. The authors have requested that Table S1 be updated and the caption of Figure 16 be replaced with the updated version below: "Figure 16: SEC chromatogram of mAb H CaptoButyl load material (orange) and CaptoButyl eluate (blue) (left) and a zoomed in view of the HMW and LMW region (right). Absorbance was measured at 280 nm."In the article (Nolan et al. 2024)

These corrections do not affect the conclusions of the study. The authors apologize for these errors.

The supplementary file has been updated.

The original article has been corrected.

Updated Fig. 16 Caption: 

SEC chromatogram of mAb H CaptoButyl load material (orange) and CaptoButyl eluate (blue) (left) and a zoomed in view of the HMW and LMW region (right). Absorbance was measured at 280 nm.
