# Supplementary figures and images for: Modeling the behavior of monoclonal antibodies on hydrophobic interaction chromatography resins
Source: Bioresour Bioprocess. 2024 Feb 15;11(1):25. doi: 10.1186/s40643-024-00738-8 (PMC10991917; doi:10.1186/s40643-024-00738-8)

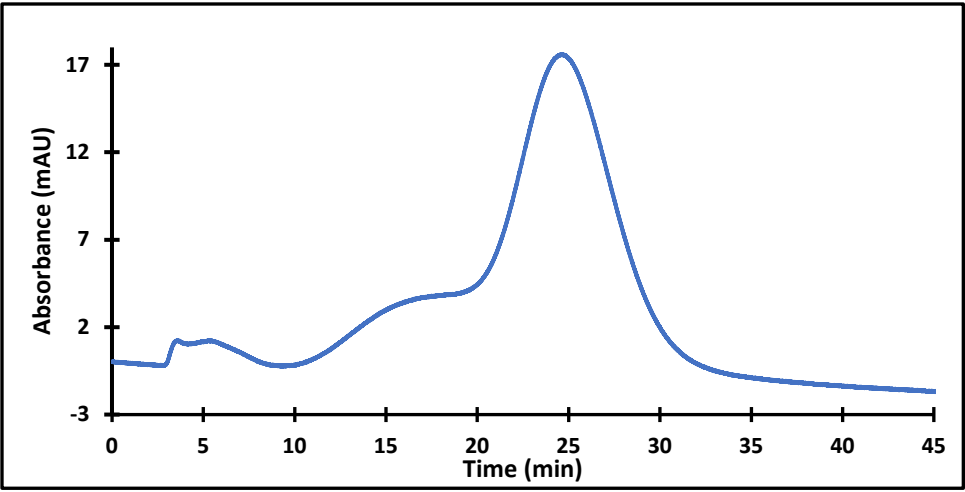

Supplement: Supplementary file 1 — Additional file 1: Figure S1. Chromatogram for the injection of 50 μL 20 mg/mL 150 kDa Dextran on a TOYOPearl Phenyl resin using a 25 mM Sodium Citrate, 5 % Isopropanol mobile phase. Dextran exhibits strong retention on TOYOPearl Phenyl resin under low salt and mild organic phase conditions. Absorbance was measured at 260 nm. [file 40643_2024_738_MOESM1_ESM.pdf]

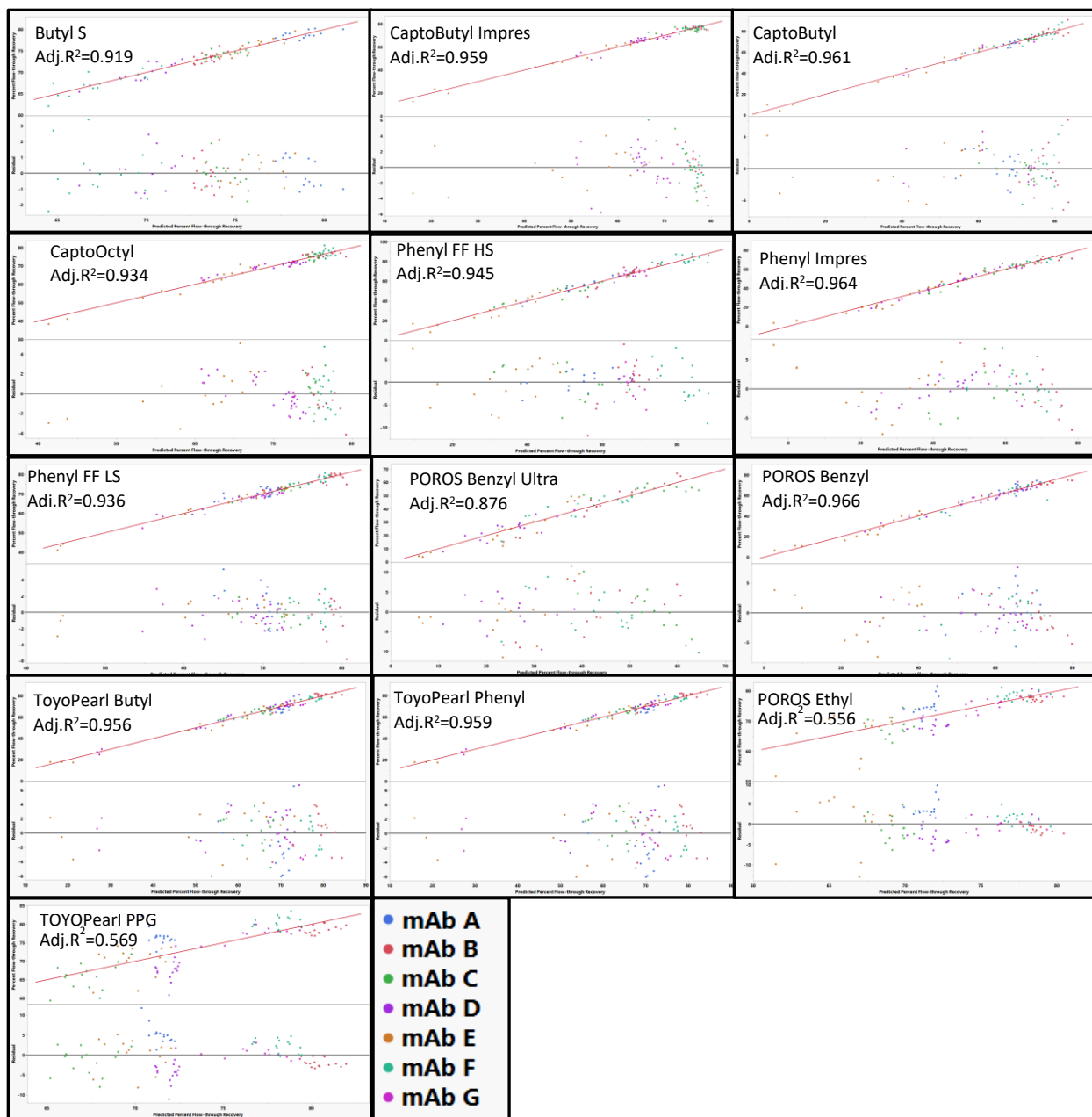

Supplement: Supplementary file 2 — Additional file 2: Figure S2. Best Actual vs Predicted and Residual plots for models predicting HMW Clearance for each resin when using RSH and CZE elution time at pH 5 as mAb descriptors. The identity line is shown in red as a reference. MAb A was an outlier on CaptoButyl ImPres and Butyl S, mAb C was an outlier on POROS Benzyl and POROS Benzyl Ultra, mAb D was an outlier on CaptoButyl and Phenyl HS and were excluded from the model. [file 40643_2024_738_MOESM2_ESM.pdf]
