# Supplementary material for: Modeling the behavior of monoclonal antibodies on hydrophobic interaction chromatography resins
Source: Bioresour Bioprocess. 2024 Feb 15;11(1):25. doi: 10.1186/s40643-024-00738-8 (PMC10991917; doi:10.1186/s40643-024-00738-8)
Supplement: Supplementary file 3 — Additional file 3: Table S1. Properties of the HIC resins used in this study. Particle size is the average particle size as reported by the vendor. [file 40643_2024_738_MOESM3_ESM.docx]

**Table 1**: Results of log k vs log k analysis for selected HIC resins. Hydrophobicity is the slope of the line resulting from log k vs log k plot when plotted against TOYOPEARL Phenyl.

| **Resin** | **Hydrophobicity** | **R^2^** | ***ζ-potential* pH 5 (mV)** | ***ζ-potential* pH 6 (mV)** | ***ζ-potential* pH 7 (mV)** |
| --- | --- | --- | --- | --- | --- |
| TOYOPEARL Phenyl | 1 | 1 | -0.12 | -1.02 | -3.80 |
| TOYOPEARL Butyl | 0.89 | 0.92 | -2.66 | -5.75 | -7.01 |
| POROS Benzyl | 0.78 | 0.92 | -12.98 | -12.99 | -14.51 |
| CaptoPhenyl Impres | 0.69 | 0.96 | -14.31 | -14.88 | -15.25 |
| CaptoButyl | 0.47 | 0.92 | -6.75 | -5.78 | -7.27 |
| CaptoButyl Impres | 0.48 | 0.91 | -3.50 | -5.51 | -7.88 |
| TOYOPEARL PPG | 0.45 | 0.87 | -1.53 | -3.99 | -7.21 |
| Butyl S FF | 0.38 | 0.82 | -0.78 | -1.58 | -1.04 |
| Phenyl FF (LS) | 0.32 | 0.89 | -1.43 | -1.49 | -3.06 |
| POROS Ethyl | 0.27 | 0.91 | -13.34 | -16.61 | -22.38 |
| CaptoOctyl | 0.20 | 0.84 | -6.72 | -6.83 | -7.85 |
| Phenyl FF (HS) | N/A | N/A | -9.72 | -8.56 | -8.54 |
| POROS Benzyl Ultra | N/A | N/A | 1.66 | -1.03 | -3.87 |
|  |  |  |  |  |  |

^a^ Polystyrene Divinyl Benzene

^b^ Polypropylene Glycol
